# The potential role of the extracellular matrix in the activity of trabectedin in UPS and L-sarcoma: evidences from a patient‐derived primary culture case series in tridimensional and zebrafish models

**DOI:** 10.1186/s13046-021-01963-1

**Published:** 2021-05-11

**Authors:** Alessandro De Vita, Federica Recine, Giacomo Miserocchi, Federica Pieri, Chiara Spadazzi, Claudia Cocchi, Silvia Vanni, Chiara Liverani, Anna Farnedi, Francesco Fabbri, Valentina Fausti, Roberto Casadei, Francesca Brandolini, Giorgio Ercolani, Davide Cavaliere, Alberto Bongiovanni, Nada Riva, Lorena Gurrieri, Giandomenico Di Menna, Sebastiano Calpona, Silvia Angela Debonis, Laura Mercatali, Toni Ibrahim

**Affiliations:** 1 Osteoncology and Rare Tumors Center, IRCCS Istituto Romagnolo Per Lo Studio Dei Tumori (IRST) “Dino Amadori”, Meldola, Italy; 2 Medical Oncology Unit, San Camillo de Lellis Hospital, Rieti, Italy; 3 Pathology Unit, Morgagni-Pierantoni Hospital, Forlì, Italy; 4 Biosciences Laboratory, IRCCS Istituto Romagnolo Per Lo Studio Dei Tumori (IRST) “Dino Amadori”, Meldola, Italy; 5 Orthopedic Unit, Morgagni-Pierantoni Hospital, Forlì, Italy; 6 General and Oncologic Surgery Unit, Morgagni-Pierantoni Hospital, Forlì, Italy; 7 Department of Medical and Surgical Sciences - DIMEC, University of Bologna, Bologna, Italy

**Keywords:** Trabectedin, Extracellular matrix, Undifferentiated pleomorphic sarcoma and L-sarcoma, Patient‐derived primary cultures, 3D scaffold, Chemotherapy

## Abstract

**Background:**

Soft tissue sarcomas (STS) are a rare group of solid neoplasm including among others liposarcoma, leiomyosarcoma (L-sarcoma) and undifferentiated pleomorphic sarcoma (UPS) entities. The current first-line treatment is represented by anthracycline based- regimens, second-line may include trabectedin. Currently the activity of trabectedin and its mechanism of action is not completely elucidated.

**Methods:**

Taking the advantages of our 3D patient-derived primary culture translational model we performed genomic-, chemobiogram, proteomic- and in vivo analysis in a UPS culture (S1). Furthermore pharmacological profiling of a UPS and L-sarcoma patient-derived case series and in silico analysis were carried out.

**Results:**

Trabectedin exhibited an increased activity in 3D respect to 2D cultures suggesting an extracellular matrix (ECM) and timp1 involvement in its mechanism of action. Moreover 3D S1 xenotranspanted zebrafish model showed an increased sensitivity to trabectedin. Finally the results were further validated in a UPS and L-sarcoma case series.

**Conclusions:**

Taken together these results confirmed the activity of trabectedin in these STS histotypes. Moreover the data underline the ECM involvement in the cytotoxic effect mediated by trabectedin and could open the door for researches aimed to focus on the patient setting that could benefit from this agent.

**Supplementary Information:**

The online version contains supplementary material available at 10.1186/s13046-021-01963-1.

## Background

The landscape of Soft Tissue Sarcoma (STS) consists in a variety of entities of mesenchymal origin with a 1 % incidence among all adult cancers [1]. Till date, the World Health Organization (WHO) classification system identifies the existence of over 80 different histologic subtypes [2]. Among these, the most frequent STS in adults include: liposarcoma (LPS), leiomyosarcoma (LMS) and undifferentiated pleomorphic sarcoma (UPS) accounting 15 %, 11% and 5 %, respectively. Although they exhibit different biological and clinical heterogeneity, LPS and LMS are usually grouped under the name of L-sarcomas [3, 4].

Chemotherapy represents the standard clinical care in metastatic setting with very poor outcomes. Antineoplastic agents routinely administered as first-line chemotherapy include anthracyclines [5]. A variety of second-line options may be considered with no optimal standard sequential therapy established [3].

Trabectedin has been approved for the treatment of advanced STS patients following failure of first-line chemotherapy or as first-line treatment for patients unfit for anthracycline-based treatment [6].

This DNA-binding compound exhibits a pleiotropic antitumor activity and an indirect immunologic and antiangiogenetic effect involving the tumor microenvironment [7]. Although different translational and clinical studies focused on the role of trabectedin in STS patients were performed, its mechanism of action has not completely elucidated. In this regard, molecular evidences suggest that it acts via the binding of the DNA minor groove leading to the DNA double-helix distortion and breakage [8–10]. In addition to these cytotoxic effects, trabectedin has a role in modulating the tumor microenvironment and some evidences tend to identify this as the most important part of its therapeutic effect [11].

To explore the mechanisms behind antineoplastic effects of trabectedin in UPS and L-sarcomas we took the advantages of our 3D collagen-based scaffold culture system and zebrafish model combined with the use of patient-derived primary cultures. This prospective study aimed to shed the light on the activity of trabectedin in UPS and L-sarcoma patients, and potentially to better define optimal treatment strategies for STS patients in clinical setting.

## Materials and methods

### Case series

The study involved ten UPS and L-sarcomas patients surgically treated by experienced orthopedic and oncologic surgeons. The explanted tumor masses were analyzed by a sarcoma pathologist and processed within 3 h of surgical resection.

### Ethical statement

The study protocol was approved by IRST-Area Vasta Romagna Ethics Committee (approval no. 4751, 31 July 2015). All the procedures were performed in accordance with GCP and Helsinki declaration. All the eligible participants gave written informed consent to take part in the study.

### Next-generation sequencing

RNA isolation, purification and quality check was performed following the manufacturer’s instructions. NGS analysis was carried out as previously reported [12]. Further information are available in Appendix 2: Supplementary methods.

### Collagen-based scaffold synthesis

The collagen scaffolds were synthesized as previously described [13–15]. Briefly, a 1 %-wt suspension of an insoluble bovine type I collagen was prepared in 0.05 M of acetic acid solution. The material was cross-linked through a 1 wt% BDDGE solution. This suspension was mixed, frozen and then freeze-dried for 24 h. The obtained scaffolds were sterilized with ethanol 70 % for 1 h and washed with PBS before using in cell culture.

### Isolation of patient-derived UPS and L-sarcoma cells

Patient-derived primary cultures were established as previously described [16, 17]. The isolated primary cells were seeded in standard monolayer cultures at a density of 80.000 per cm^2^ or in collagen-based scaffolds at a density of 500.000 cells/ 57 mm^3^ and maintained in complete DMEM medium. Seeding on 3D collagen-based scaffolds was achieved as previously described [18, 19]. All the experiments were conducted using low-passage and proliferating primary cultures.

### Gene expression profiling

mRNA isolation was obtained using TRIzol Reagent (Invitrogen) following the manufacturer’s instructions. iScript cDNA Synthesis Kit (BioRad) was used to reverse transcribe 500 ng of extracted RNA. Real-Time PCR was performed on the 7500 Real-Time PCR System using the TaqMan gene expression assay mix (Applied Biosystems). A total volume of 20 µL containing 2 µL of Taqman Universal PCR Master Mix (Applied Biosystems) and 2 µL of cDNA was used for the amplification. The amount of transcripts was normalized to the reference genes with the 2 ΔΔCt method and expressed as n-fold mRNA levels relative to a calibrator (see complete list of genes in Supplementary Table S1). RNA extracted from the tumor tissue was used as calibrator.

### Pharmacological and proteomic profiling

Primary tumor cells seeded both in standard plates and in 3D scaffolds were allowed to recover for 3 days and then were exposed to drugs. The regimens were selected according to peak plasma concentration of each drug from pharmacokinetic clinical data and they were: ifosfamide (IFO), epirubicin (EPI), combination of IFO + EPI, doxorubicin (DOXO), trabectedin (TRABE), eribulin (ERI), dacarbazine (DACA) and lenvatinib (LENVA) (see complete list of chemotherapeutic in Supplementary Table S2). Cell viability percentage was assessed, as previously reported [20], through MTT reduction assay (Sigma Aldrich) after drug exposure for 72 h. Experiments were performed twice (see complete list of antibody in Supplementary Table S3).

### Zebrafish xenograft

AB wild type zebrafish embryos (Appendix 2: Supplementary methods) obtained and stored according to Kimmel et al. [21] were dechorionated at 48 h post fertilization (hpf). Cells were labeled with a red fluorescent dye (CellTracker™ CM-DiI, Invitrogen) at the concentration of 2.5 × 10^5^/µl. 300/500 cells were injected in the yolk sack of 48 hpf embryos. Grafted embryos with 2D or 3D cultured primary cells were divided in two groups that were treated with TRABE (PharmaMar) or no treated (20 zebrafish embryos per condition). Embryos were incubated at 32 °C for 72 h. Image of tumor area were performed using a fluorescence stereomicroscope (Nikon SMZ 25 equipped with NIS Elements software).

### Statistical analysis

Two or three independent replicates were performed for each experiment. Data are presented as mean ± standard deviation or standard error, with n indicating the number of replicates. Differences between groups were assessed by a two-tailed Student’s t-test and accepted as significant at *p* < 0.05.

## Results

### Patients

Clinical pathological characteristics of patients are reported in Table 1. Patient clinical history is available in Supplementary data information.

**Table 1:** Clinical pathologic characteristics of UPS and L-sarcoma patients enrolled in the study

Patient	Sex	Age at surgery (years)	site	Size of major axis (cm)	Histological subtype	IHC analysis	Molecular cytogenetic analysis	Grade	Tumor	Surgical margins	Radiotherapy post-surgery	Chemotherapy post-surgery	Follow up (months)
S1	Female	81	lower arm	13	UPS	na	MDM2/ CEP12 = 1 FUS (16p11)˂10 %	G3	Primary tumor	R1	Yes	No	26
S2	Female	83	gluteus	2.5 ;1	DDLPS	na	MDM2/ CEP12 = 2	G3	Local recurrence	R1	Yes	No	46
S3	Female	72	lower arm	15	DDLPS	na	MDM2/ CEP12 = 2 FUS (16p11)˂10 %	G3	Local recurrence	R0	No	No	14
S4	Female	61	thigh	20	ALT/WDLPS	na	MDM2/ CEP12 = 2	G3	Primary tumor	R0	No	No	11
S5	Female	77	retroperitoneum	10	LMS	SMA + desmin +	MDM2/ CEP12 = 1	G2	Primary tumor	R1	No	No	10
S6	Female	71	retroperitoneum	10	ALT/WDLPS	na	MDM2/ CEP12 = 2	G1	Primary tumor	R1	No	No	12
S7	Male	56	trunk	15	ALT/WDLPS	na	MDM2/ CEP12 = 2	G1	Primary tumor	R0	No	No	2
S8	Female	78	thigh	6	UPS	SMA + CD31 – CD34 – S100 –cytokeratins- desmin - CD117 -	MDM2/ CEP12 = 1	G3	Primary tumor	R0	Yes	No	9
S9	Female	57	abdomen	9	DDLPS	na	MDM2/ CEP12 = 2	G3	Primary tumor	R1	No	No	6
S10	Female	64	thigh	7	PLS	na	MDM2/ CEP12 = 1	G3	Primary tumor	R1	No	Yes	4

*IHC *immunohistochemical analysis, *n/a *not applicable, *SMA *smooth muscle actin, *R0 *microscopically margin-negative resection, *R1 *microscopically margin-positive resection

### Establishment of a 3D patient-derived culture of high-grade UPS

**Fig. 1:**
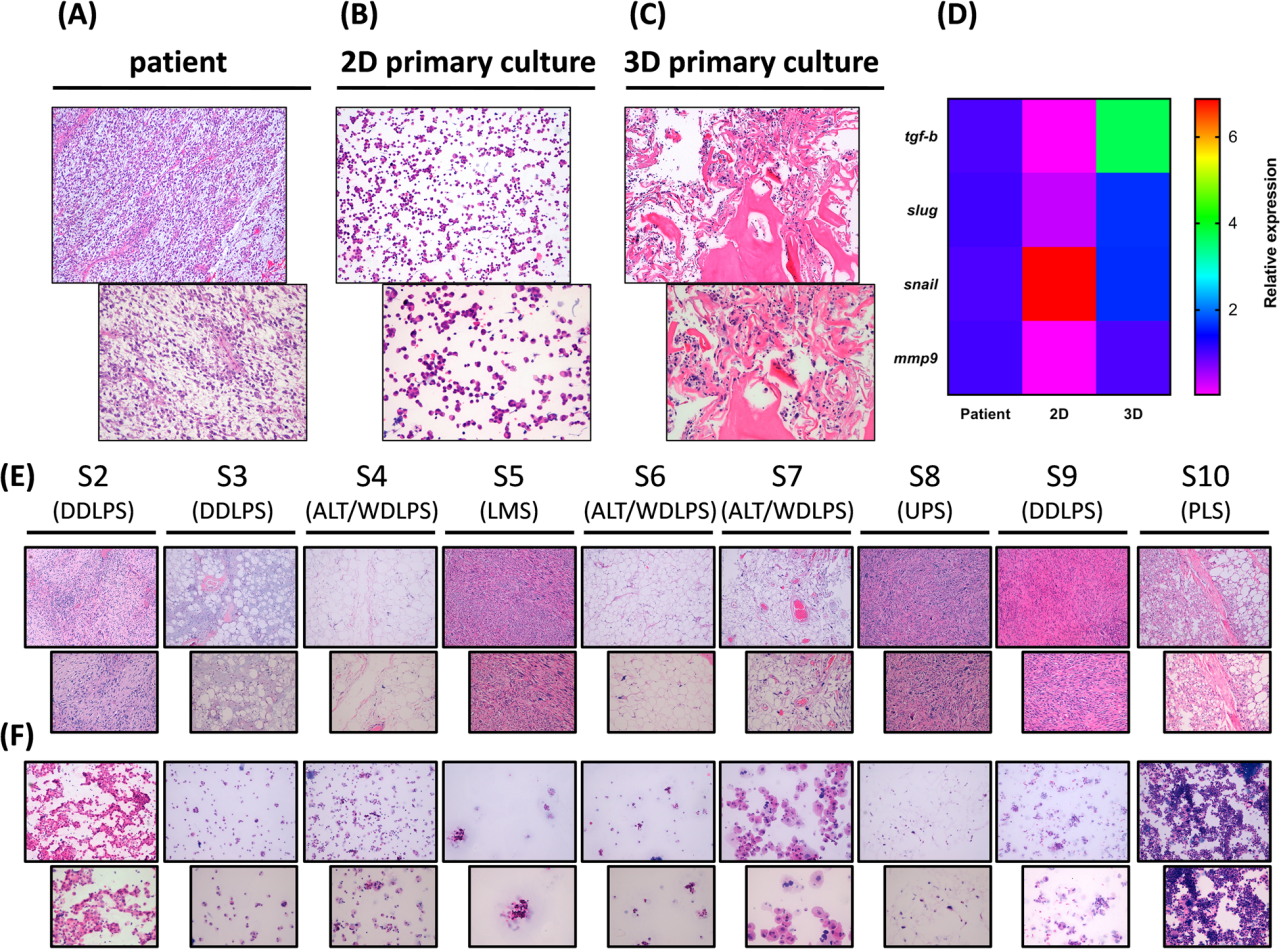
**a** hematoxylin and eosin staining of the patient surgical specimen showing high-grade polymorphic UPS cells (light blue stroma) infiltrating necrotic tissue, 10 x and 20 x magnification. **b** hematoxylin and eosin staining of the cytospunned tumor cells from the patient-derived UPS primary culture S1, 10 x and 20 x magnification. **c** hematoxylin and eosin staining of the cytospunned tumor cells (light blue spots) from the patient-derived UPS primary culture S1 cultured within 3D collagen-based scaffold system, 10 x and 20 x magnification. **d** Heat map comparisons of the relative gene expression of selected tumor-associated markers tgf-b, slug, snail, mmp9 between UPS patient surgical specimen, S1 patient-derived 2D standard monolayer primary culture and S1 patient-derived 3D primary culture system. **e** hematoxylin and eosin staining of the patient surgical specimen showing low and high grade UPS and L-sarcoma (light blue stroma) infiltrating adipose tissue, 10x and 20× magnification. **f** hematoxylin and eosin staining of the cytospunned tumor cells of patient-derived primary cultures UPS and L-sarcoma case series, 10 x and 20 x magnification

The diagnosis of the surgically-resected tumor tissue (Fig. 1a and Supplementary Fig. S1) was high-grade polymorphic UPS. Molecular analysis of both MDM2 gene amplification and FUS-CHOP rearrangement were negative. NGS analysis of a STS fusions associated gene panel yielded no fusion call (Supplementary Fig. S2). Cytomorphologic features analysis of 2D and 3D primary cells confirmed the establishment of a patient-derived high grade UPS culture with a proportion of 20 and 45 % UPS cells respectively (Fig. 1b-c). In 2D the tissue-like organization was completely lost as in part the tumor cell morphology of the patient counterpart. In contrast the 3D model preserved a tissue-like organization with cell morphological features and distribution similar to that of tumor sample. Moreover a UPS and L-sarcoma primary culture case series was established for pharmacological profiling (Fig. 1e-f and Appendix 3: Supporting information).

### Preservation of gene expression in 3D culture model

Markers involved in tumor aggressiveness were evaluated (Fig. 1d). Our model was able to preserve the expression of some markers belonging to several tumor-associated pathways respect to 2D culture. In particular *tgf-b* expression, was 33-fold lower than that of control in 2D while was 2.4 fold-higher increase in 3D. *slug* was 3.2-fold lower than that of control in 2D and 0.8-fold increase in 3D. *snail* was 5.4 fold-higher increase in 2D respect to that of the control and 0.5 in 3D compared to that of the control. Finally *mmp9* was 25-fold lower than that of control in 2D and 0.1-fold lower in 3D.

### Pharmacological profile of patient-derived high-grade UPS primary culture: 2D versus 3D

The role of chemotherapy in the UPS patient-derived S1 culture was investigated. S1 cultured in 2D and in 3D was exposed to IFO, EPI, to the combination of IFO and EPI and DOXO. Moreover, the efficacy of some second-line treatments as TRABE, ERI and DACA were evaluated. Finally, the activity of LENVA, was assessed.

**Fig. 2:**
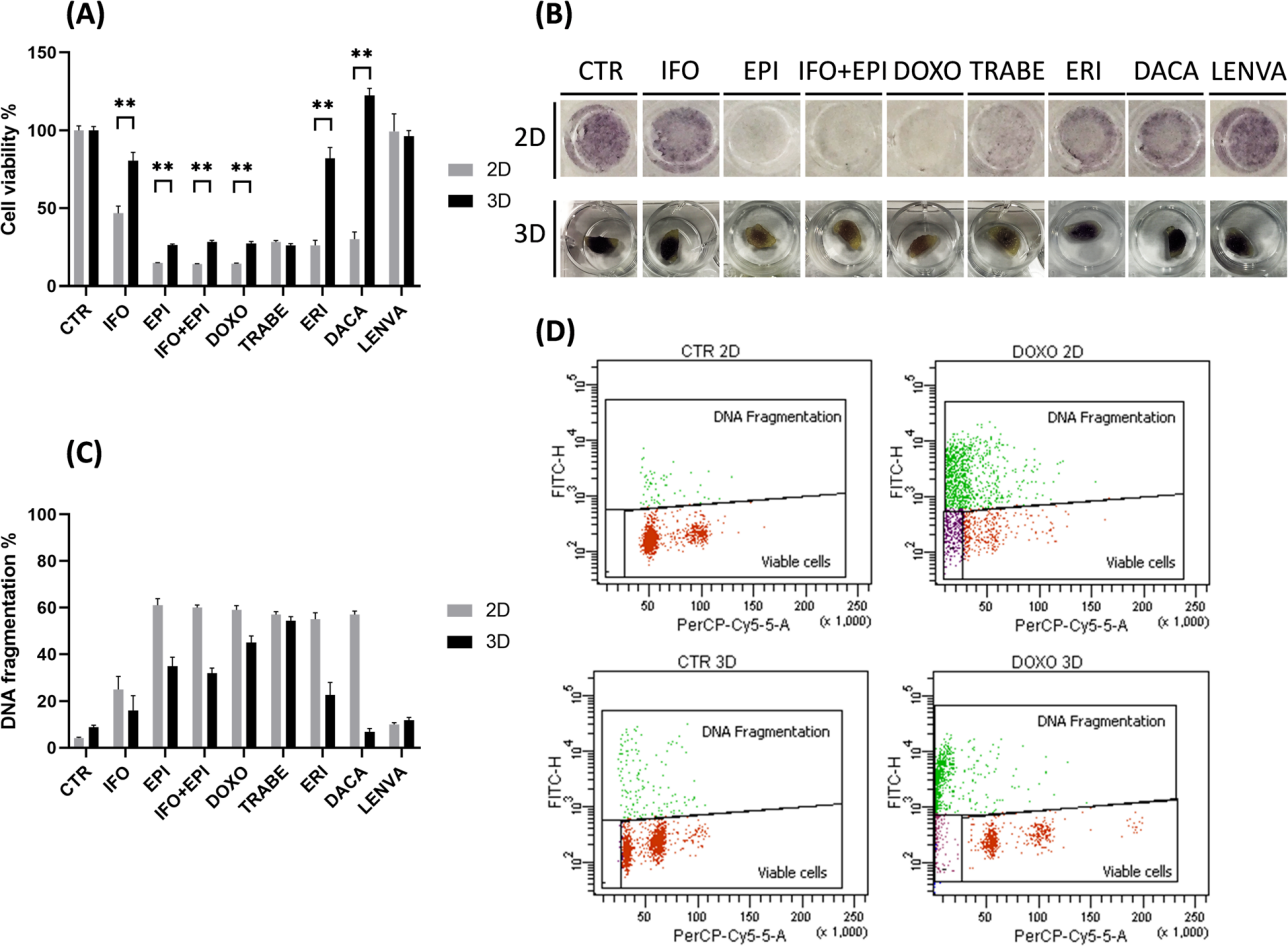
**a** Chemobiogram analysis of S1 primary culture seeded in 2D and 3D-collagen based scaffold and exposed to chemotherapeutics agent, untreated cells were used as control. **b** Representative images of 2D and 3D-collagen based scaffold S1 primary culture exposed to the tested drugs. **c** DNA fragmentation analysis expressed as apoptotic cells % of 2D and 3D-collagen based scaffold S1 primary culture exposed to the tested drugs. **d** Representative images (dot plot) of DNA fragmentation analysis obtained thought flow cytometry

UPS cells cultured in 2D showed a survival of: 46 % with IFO, 14 % with anthracyclines-based regimens, 28 % with TRABE, 26 % with ERI, 30 % with DACA and 99 % with LENVA (Fig. 2a-b).

UPS cells cultured in 3D showed a survival of: 80 % with IFO, 27 % with anthracyclines-based regimens, 25 % with TRABE, 82 % with ERI, DACA did not affected the survival and 96 % with LENVA (Fig. 2 a-b).

Significant differences between 2D and 3D treatments were detected for all the tested drugs excluding TRABE and LENVA.

Next, the cell viability results were confirmed through a live-dead staining using flow cytometry analysis (Fig. 2c-d).

A higher sensitivity to treatments in 2D compared to 3D was observed in all treatment conditions while this was not evident with TRABE.

### Chemotherapy induces apoptosis through the caspase-dependent apoptotic pathway in 3D high-grade UPS primary culture

**Fig. 3:**
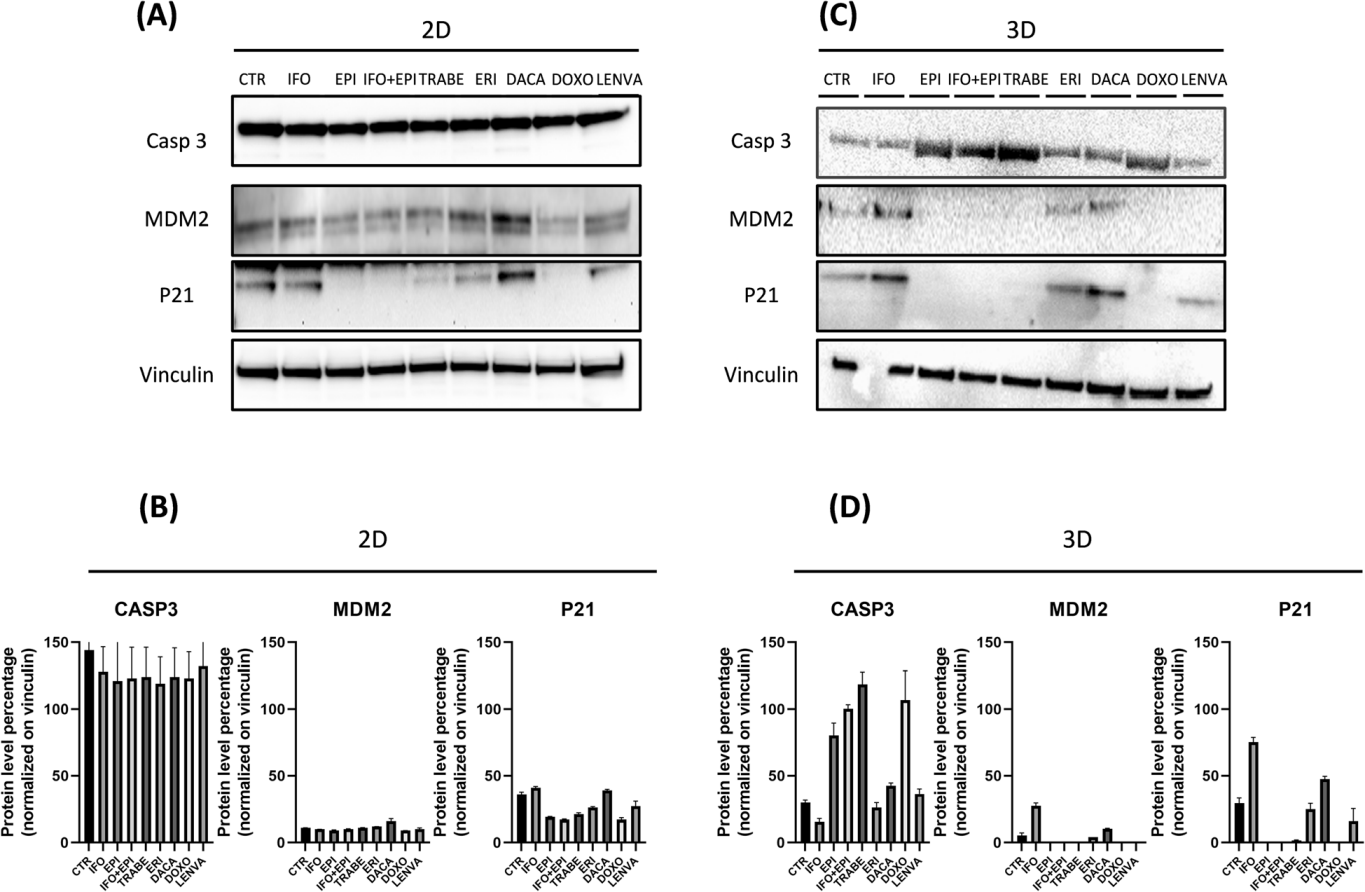
**a** Proteomic analysis of apoptotic- and anti-apoptotic-related proteins in 2D S1 primary culture exposed to the tested drugs. **b** Densitometric analysis of protein bands % normalized on the housekeeping vinculin. **c** Proteomic analysis of apoptotic- and anti-apoptotic-related proteins in 3D-collagen based scaffold S1 primary culture exposed to the tested drugs. **d** Densitometric analysis of protein bands % normalized on the housekeeping vinculin

To further determine how chemotherapy induces cytotoxic effect in S1, the expression levels of apoptotic- and anti-apoptotic-related proteins were assessed (Fig. 3). Casp-3 was upregulated in the negative control and in all treatment groups. In this regard no differences in the level expression between the control and treatment groups were observed in 2D (Fig. 3a-b), while in 3D the highest expression was observed in TRABE followed by the anthracycline-based regimens (Fig. 3c-d). The chemotherapy resistance related-gene MDM2 was upregulated in 2D in all treatment groups (Fig. 3a-b), while was downregulated with anthracyclines-based regimens, TRABE and LENVA and upregulated with IFO, ERI and DACA in 3D (Fig. 3c-d). Finally the MDM2 expression was also detected in the control in both 2D and 3D which is in line with patient diagnosis. The anti-apoptotic protein p21 was upregulated in the control and in all treatment groups in 2D and after IFO, ERI, DACA and LENVA treatments in 3D.

### Metallopeptidase inhibitor timp1 seems associated to the activity of trabectedin in 3D

**Fig. 4:**
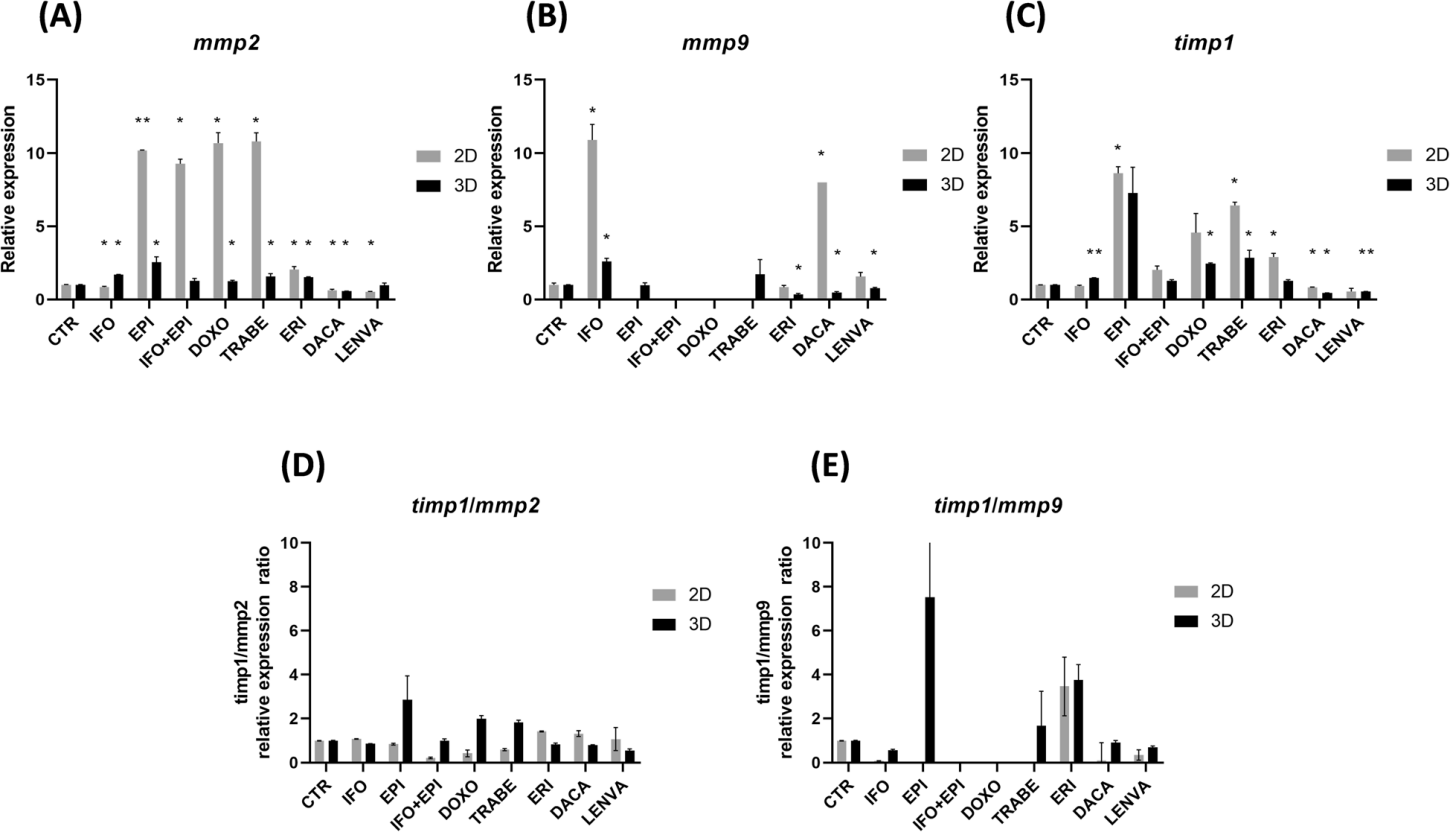
**a**-**c** Relative expression of ECM-associated genes in 2D and 3D-collagen based scaffold S1 primary culture. **d** timp1/mmp2 relative expression ratio genes in 2D and 3D-collagen based scaffold S1 primary culture. **e** timp1/mmp9 relative expression ratio genes in 2D and 3D-collagen based scaffold S1 primary culture

To better elucidate if the presence of the ECM components could affect the mechanism of action of the tested drugs, ECM-associated markers were evaluated in S1 cultured in 2D and 3D and compared to the expression of untreated cells. The expression of matrix modifying enzymes *mmp2* and *mmp9* and their inhibitor *timp1* was analyzed (Fig. 4a-c).

Gene expression analysis of *mmp2* in 2D was significantly upregulated for all the tested drugs, meanwhile *mmp2* expression in 3D was significantly upregulated for IFO, EPI, DOXO, TRABE, ERI and DACA. A not significant upregulation was detected with IFO + EPI and LENVA.

Gene expression analysis of *mmp9* in 2D was: significantly upregulated for IFO, DACA and LENVA, not detected for anthracyclines based-therapy and TRABE, meanwhile *mmp9* expression in 3D was significantly upregulated for IFO, not detected for IFO + EPI and DOXO.

Gene expression analysis of *timp1* in 2D was: significantly upregulated for anthracyclines based-therapy, TRABE, ERI, meanwhile *timp1* in 3D was significantly upregulated for IFO, DOXO, TRABE, DACA and LENVA. A not significant upregulation was detected with EPI, IFO + EPI, ERI.

Since *timp1* is an inhibitor of *mmps* the *timp1*/*mmp2* and *timp1*/*mmp9* expression ratios in 2D and 3D were analyzed (Fig. 4d-e).

*Timp1*/*mmp2* relative expression ratio in 2D was: ≥ 1.0 for IFO, ERI, DACA, LENVA; < 1.0 for anthracyclines based-therapy, TRABE; meanwhile in 3D was ≥ 1.0 for anthracyclines based-therapy, TRABE; < 1.0 for IFO, ERI, DACA, LENVA.

*Timp1*/*mmp9* relative expression ratio in 2D was: ≥ 1.0 for ERI; < 1.0 for IFO, DACA, LENVA; undetermined for anthracyclines based-therapy and TRABE; meanwhile in 3D was ≥ 1.0 for EPI, TRABE, ERI; < 1.0 for IFO, DACA and LENVA; undetermined for IFO + EPI and DOXO.

### UPS primary cells cultured within collagen-based scaffold are more susceptible to trabectedin activity in zebrafish model

**Fig. 5:**
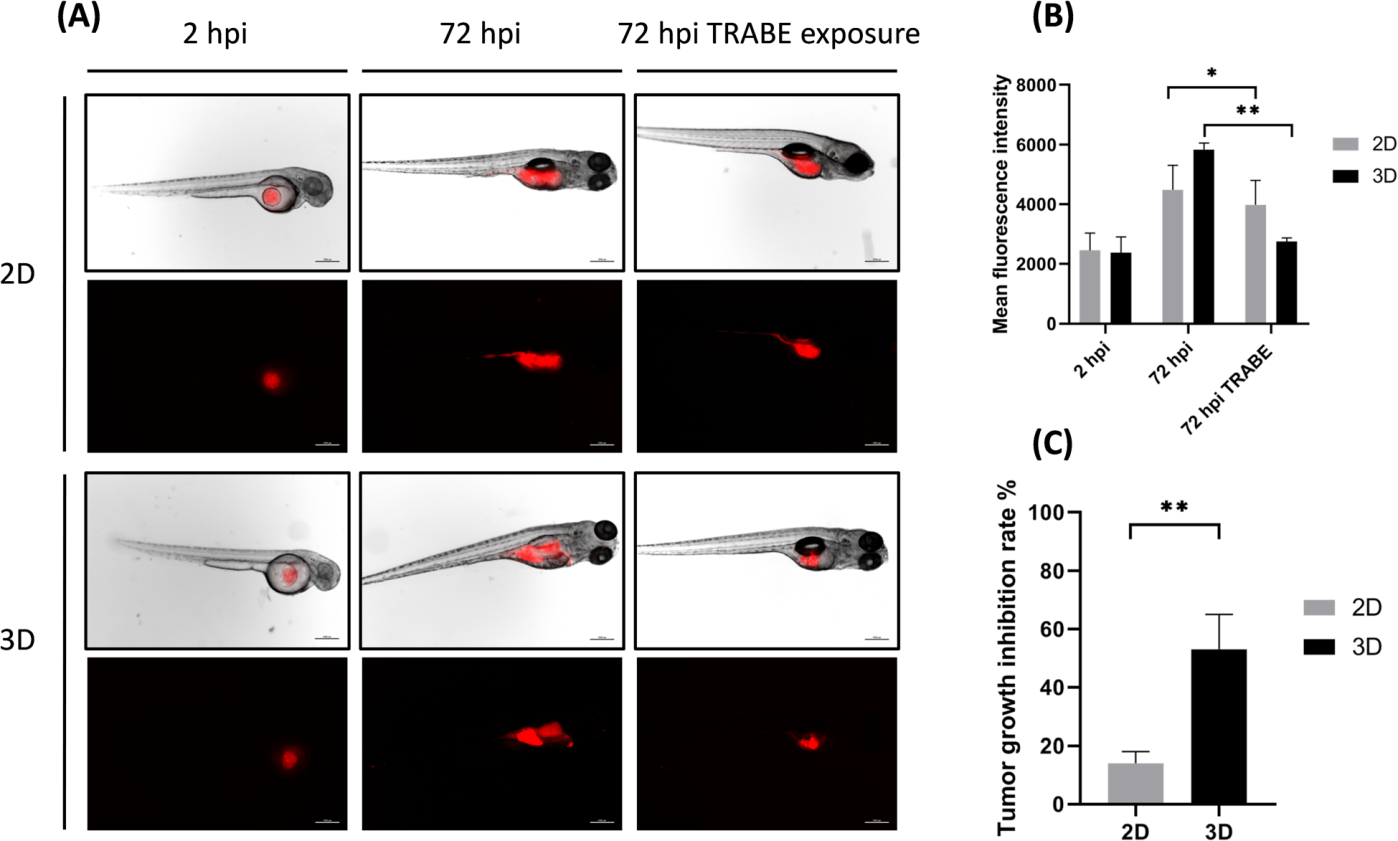
**a** Representative fluorescence microscopy images of zebrafish embryos xenotrasnplanted with S1 cultured in standard monolayer culture (2D) and within 3D collagen-based scaffold (3D). Images of embryos untreated at 2 and 72 h post injection and exposed to trabectedin at 72 h post injection, scale bar 1000 μm. **b** Mean fluorescence signal of 2D and 3D xenotransplanted embryos, arbitrary units. **c** Tumor growth inhibition rate between 2D and 3D groups

In order to consolidate the data observed *in vitro*, S1 cultured in 2D and in 3D were xenotrasplanted in zebrafish embryos (Supplementary Fig. S3). The engraftment was successfully achieved in both conditions. Tumor growth imaged at 2 and 72 h post injection (hpi) showed equivalent fluorescence signals with both conditions at 2 hpi while at 72 hpi an increased signal of 3D condition was detected compared to 2D (Fig. 5a-b). Moreover *in vivo* cancer cells proliferation was significantly suppressed after 72h TRABE treatment in both 2D and 3D culture systems with the greater tumor growth inhibition rate in 3D (14 % for 2D and 53 % for 3D, Fig. 5c) compared to untreated group. Among all treated zebrafish embryos, the mortality was of 12.5 % and 4 embryos presented severe abnormalities (Supplementary Fig. S4) and were excluded from the study. No morphological abnormities were detected in the untreated group.

The above observation prompted us to validate these data in a case series of STS patient-derived primary cultures.

### Trabectedin exhibited a higher activity in 3D and a comparable effect to anthracyclines in a UPS and L-sarcoma primary culture case series

**Fig. 6:**
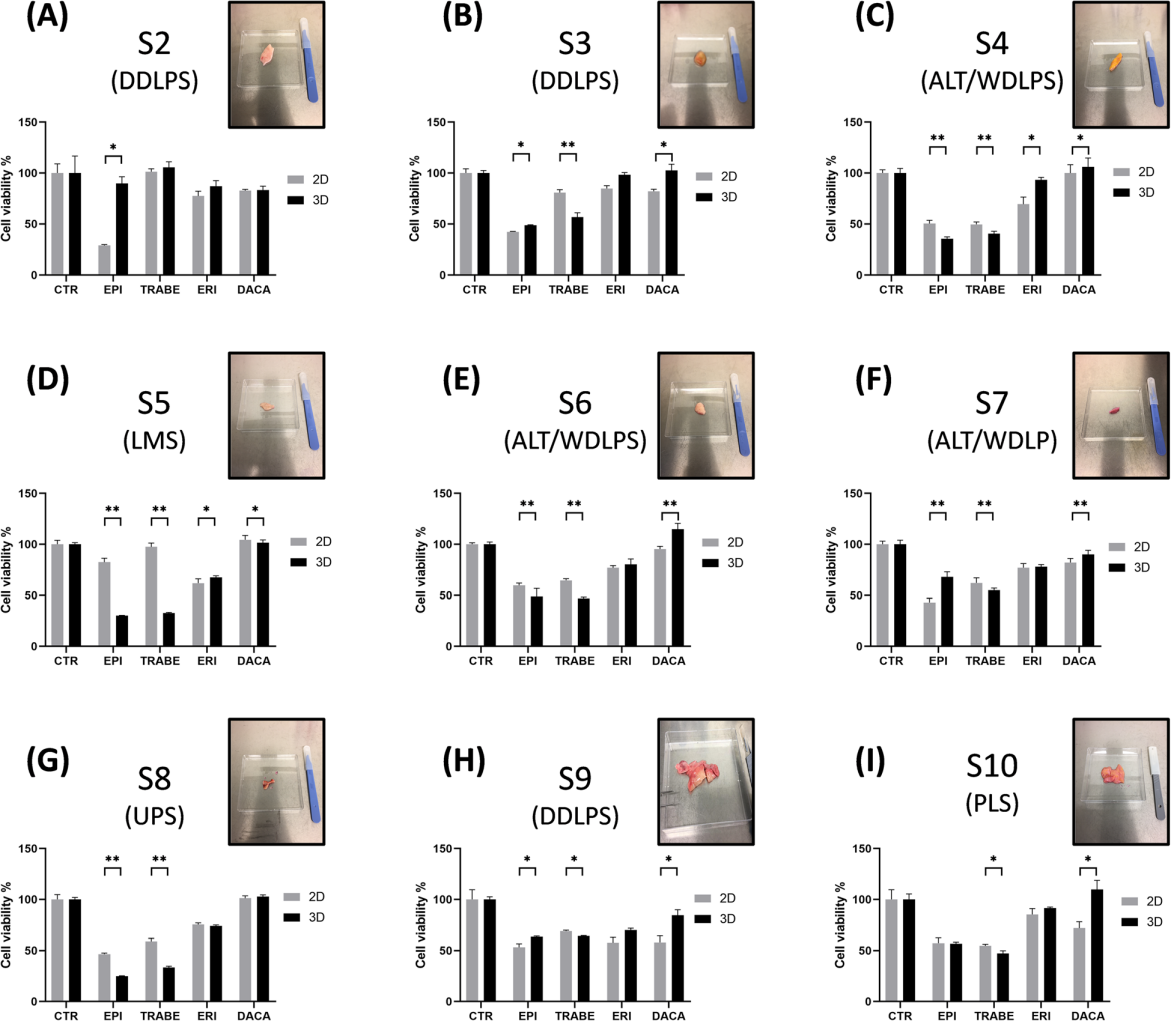
Pharmacological profile of 2D and 3D-collagen based scaffold UPS and L-sarcoma primary culture case series. Primary cells were exposed to selected first- and second- line treatments (EPI, TRABE, ERI, DACA) for STS. Images of the surgical specimens used for the establishment of primary cultures are reported (**a**) S2 DDLPS. **b** S3 DDLPS. **c** S4 ALT/WDLPS. **d** S5 LMS. **e** S6 ALT/WDLPS. **f** S7 ALT/WDLPS. **g** S8 UPS. **h** DDLPS. **i** PLS

We investigated the activity of some first- and second- line treatments in UPS and L-sarcoma primary cultures. Established primary cultures (Fig. 1e-f and Appendix 3: Supporting information) were exposed to EPI, TRABE, ERI, DACA both in 2D and in 3D models (Fig. 6, Supplementary Fig. S5).

Among all patient the most active treatment were EPI and TRABE both in 2D and 3D (Fig. 6).

Significant increased activity in 3D with TRABE was observed for all patients excluding S2.

Significant increased activity in 3D with EPI was detected for S4-S6 and S8, and a decrease activity in S2, S3, S7, S9. No significant differences were detected in S10 patient.

No significant increased activity in 3D were observed with ERI and DACA.

## Discussion

The aim of our study was to investigate the trabectedin activity in UPS and L-Sarcoma, thus we conceived a prospective study based on the use of patient-derived primary cultures combined with 3D culture system and zebrafish model. This study was carried out from June 2016 to February 2020 enrolling ten patients affected by UPS and L-sarcoma.

Our model exhibited, as previously shown [13, 22], a higher degree of morphological and genomics similarity to patient tumor compared to 2D system. Next, chemobiogram and FACS results (Fig. 2 a-d) revealed a significant increased S1 sensitivity for all tested drugs in 2D compared to 3D, confirmed also by Casp-3 upregulation in all treatment groups in 2D (Fig. 3a-b), no differences between 2D and 3D occurred with TRABE and LENVA (Fig. 2a-d). Thus we speculate that this evidence can be related to the mechanism of action exerted by these drugs. In particular, since TRABE and LENVA interfere respectively on tumor microenvironment modulation and in the vascular endothelial formation we can hypothesize their full spectrum of activity was underestimated in *in vitro* standard culture. The most active treatments were anthracyclines based-therapy and TRABE in 3D, confirmed also through Casp-3 proteomic analysis (Fig. 3c-d). These activity results observed between anthracyclines based-therapy and TRABE could provide support to the current use in clinical practice of TRABE as an up-front in elderly and frail people [3].

Furthermore, MDM2 downregulation mediated by LENVA in 3D could provide rationale for testing this drug in MDM2 amplified STS histotypes.

Based on the above results, we hypothesized that trabectedin might affect the S1 growth through a mechanism involving ECM-associated genes including *mmps* and their inhibitor *timp1* (Fig. 4a-c). The results showed a *timp1* upregulation in both 2D and 3D especially with anthracyclines-based regimens and TRABE (Fig. 4c). The above data are consistent with previous research [23] in which trabectedin seems to promote the increase of *timp1* expression which may affect the *mmps* activity contributing to the inhibition of cell invasiveness.

In this context, while *timp1* and *mmp2* were upregulated and *mmp9* was undetectable in 2D with TRABE, all of these genes were upregulated in 3D. This data suggested a possible saturation of *timp1* mediated-inhibition mechanism of *mmps* in 3D. This could serve as an explanation of the equivalent cytotoxic activity of TRABE detected between 2D and 3D (Figs. 3 and 4). This data was not observed with other tested drugs.

Moreover, while an increase in the *timp1*/*mmp2* ratio (Fig. 4d) between 2D and 3D does not improved the sensitivity to anthracyclines-based regimen this was observed in TRABE (Fig. 2a). In particular a lower sensitivity was expected in 3D, as observed for all the other tested drugs, while a similar trend was detected. The latter result could be related to the increased *timp1*/*mmp2* ratio which could have affected the sensitivity to TRABE in 3D.

Furthermore, the higher *timp1*/*mmp9* ratio observed in 3D in EPI (Fig. 4e) compared to TRABE was not related to an increased sensitivity of EPI in 3D (26 % for EPI and 25 % for TRABE). This result supports the above observation that *timp1* could be involved with TRABE activity in 3D and not with other investigated drugs.

Indeed the obtained results are corroborated by the missing correlation of *timp1* activity with an higher sensitivity to chemotherapy in 3D with all the other tested drugs.

The *in vitro* data were further validated in *in vivo*. In particular, 3D S1 primary cells were more susceptible to TRABE also when injected into zebrafish embryos (Fig. 5c).

Previous results were validated in a UPS and L-sarcomas case series in which a significant increased activity in all 3D primary cultures was observed only with TRABE (Fig. 6). This evidence can be related to the unique trabectedin mechanism of action including a remodeling of ECM components and cells cytoskeleton. Moreover, as shown [22] our 3D model display an induction of biomarkers expression associated with STS pathogenesis (Fig. 1d) which could represent potential targets of TRABE. Furthermore TRABE could affect the expression of ECM-related genes as collagen type 1 produced by tumor cells [24, 25] with suggestions that it may reduce the high turnover of the tumor stroma. Therefore results from a chondrosarcoma model showed that trabectidin prolonged exposure determine a decrease in mRNA expression of types I and IV collagen α1 chain which may to be related to the drug resistance [8]. Finally, preclinical evidences suggested that trabectedin acts also via the inhibition of ECM degradation mediated by tumor cells causing the inhibition of cell invasiveness [23]. The above results provide support to our observations that the increased activity occurred in our study by trabectedin in 3D could be explainable by the presence of collagen type 1 compared to 2D culture.

S2 was the only primary culture among all that did not show the significant trend in favor of 3D observed with TRABE. This result is consistent with the clinical outcome observed in the patient, which showed a progression of disease during the first line with trabectedin therapy. One of the possible explanations could be related to the several local recurrences of DDLPS that could have promoted resistance of the patient to following treatments, compared to the other patients in which we tested the antitumor activity of TRABE in the primary tumor.

A similar trend was observed with EPI in only a half of analyzed cultures, showing considerable variations in the sensitivity of the three main subtypes of LPS, LMS and UPS to anthracyclines. This evidence could suggest a possible predominant cytotoxic effect mediated by EPI rather than an involvement of ECM.

Moreover the results of another DNA-binding drug as EPI suggest that the activity of TRABE in 3D is not related to the ability of our 3D model in selecting an increased tumor cell population but to the ECM involvement in the mechanism of action of this drug.

Furthermore, *in silico* analysis (Appendix 3: Supporting information) showed the upregulation of the ECM component *col1a1* in various tumors (Supplementary Fig. S6). Noteworthy, the highest expression was observed in STS followed by breast invasive carcinoma. Trabectedin has been approved for the treatment of advanced STS patients and its activity has been also showed in breast invasive carcinoma [26]. Taken together these results are supporting the ECM involvement in the activity of trabectedin. Moreover *timp1* exhibited a positive prognostic role in STS disease free survival and *mmp2* was upregulated in various tumors with the highest expression in STS (Supplementary Fig. S7) supporting their role in STS disease.

## Conclusions

In conclusion the results of this study are suggestive of the great contribution mediated by culturing models and primary cultures [27] in treatment response. On the other side our study presented several limitations. The small number of patients involved and the heterogeneity in primary cultures tumor cells may be correlated to a variability of the results. Moreover preclinical model fails to completely reproduce the spectrum of all tumor features. Another criticism is represented by the use of ifosfamide instead of its active metabolite. Our tentative to limit this bias lies on the coupling of different approaches including *in vitro* 3D system, the use of primary cultures, and an *in vivo* model.

Overall, our results are consistent with the clinical evidence that trabectedin is effective in L-sarcomas [28, 29], however some retrospective and randomized trials suggested an antitumor activity of trabectedin in non-L-sarcomas including UPS [30, 31]. In this regard, the clinical relevance of our study is represented by the potential use of trabectedin also for the STS characterized by an aggressive behavior, as UPS.

To the best of our knowledge this is the first translational work on a UPS and L-sarcoma 3D patient-derived primary cultures case series in which the role of chemotherapy and especially trabectedin was investigated. Moreover, this is the first research in which the activity of trabectedin has been analyzed *in vivo* through the use of UPS primary culture in zebrafish model.

Ongoing clinical trials are focusing on the activity of trabectedin in mono-regimen or in combination with novel drugs for STS treatment (NCT03985722, NCT02398058).

The results of this study shed the light on the potential role of ECM in the mechanism of action of trabectedin in some of most frequent STS histotypes in adults. The work underlines the involvement of this tumor microenvironment component in predicting response to trabectedin and provide the rationale for better stratifying patients which would be candidate for this drug. Further researches are needed to confirm these evidences.

## Supplementary Information

**Additional file 1: Supplementary figures.**

**Additional file 2: Supplementary methods.**

**Additional file 3: Supporting information.**

## Data Availability

The datasets generated and/or analysed during the current study are available from the corresponding author on reasonable request.

## Abbreviations

ALT/WDLPS: Atypical lipomatous tumor or well-differentiated
BDDGE: 1,4-butanedioldiglycidyl ether
casp-3: Cysteine-asparticacid protease
col1a1: Collagen type I alpha 1 chain
CT: Computed tomography
DACA: Dacarbazine
DDLPS: Dedifferentiated liposarcoma
DMEM: Dulbecco’s modified Eagle’s medium
ECM: Extracellular matrix
EPI: Epirubicin
ERI: Eribulin
FISH: Fluorescence in situ hybridization
GEPIA 2: Gene expression profiling interactive analysis 2
H&E: Hematoxylin and eosin
Hpi: Hours post injection
IFO: Ifosfamide
IFO+EPI: Ifosfamide+epirubicin
LENVA: Lenvatinib
LMS: Leiomyosarcoma
LPS: Liposarcoma
MDM2: Mouse double minute 2 homolog
MLPS: Myxoid liposarcoma
mmp2: Matrix metallopeptidase 2
mmp9: Matrix metallopeptidase 9
MRI: Magnetic resonance imaging
p21: Cyclin-dependent kinase inhibitor 1
PET: Positron emission tomography
PLS: Pleomorphic sarcoma
slug: Snail family transcriptional repressor 2
SMA: Smooth muscle actin
snail: Zinc finger protein SNAI1
STS: Soft tissue sarcoma
SUV: Standard uptake value
TAM: Tumor-associated macrophages
tcga: The Cancer Genome Atlas
tgf-b: Transforming growth factor-beta
TIMER: Tumor IMmune Estimation Resource
timp1: TIMP metallopeptidase inhibitor 1
tpm: Transcripts per million
TRABE: Trabectedin
UPS: Undifferentiated pleomorphic sarcoma
WHO: World Health Organization
