# Supplementary material for: The potential role of the extracellular matrix in the activity of trabectedin in UPS and L-sarcoma: evidences from a patient‐derived primary culture case series in tridimensional and zebrafish models
Source: J Exp Clin Cancer Res. 2021 May 11;40:165. doi: 10.1186/s13046-021-01963-1 (PMC8111914; doi:10.1186/s13046-021-01963-1)
Supplement: Supplementary file 1 — Additional file 1: Supplementary figures. [file 13046_2021_1963_MOESM1_ESM.docx]

**APPENDIX 1 - SUPPLEMENTARY FIGURES**

**
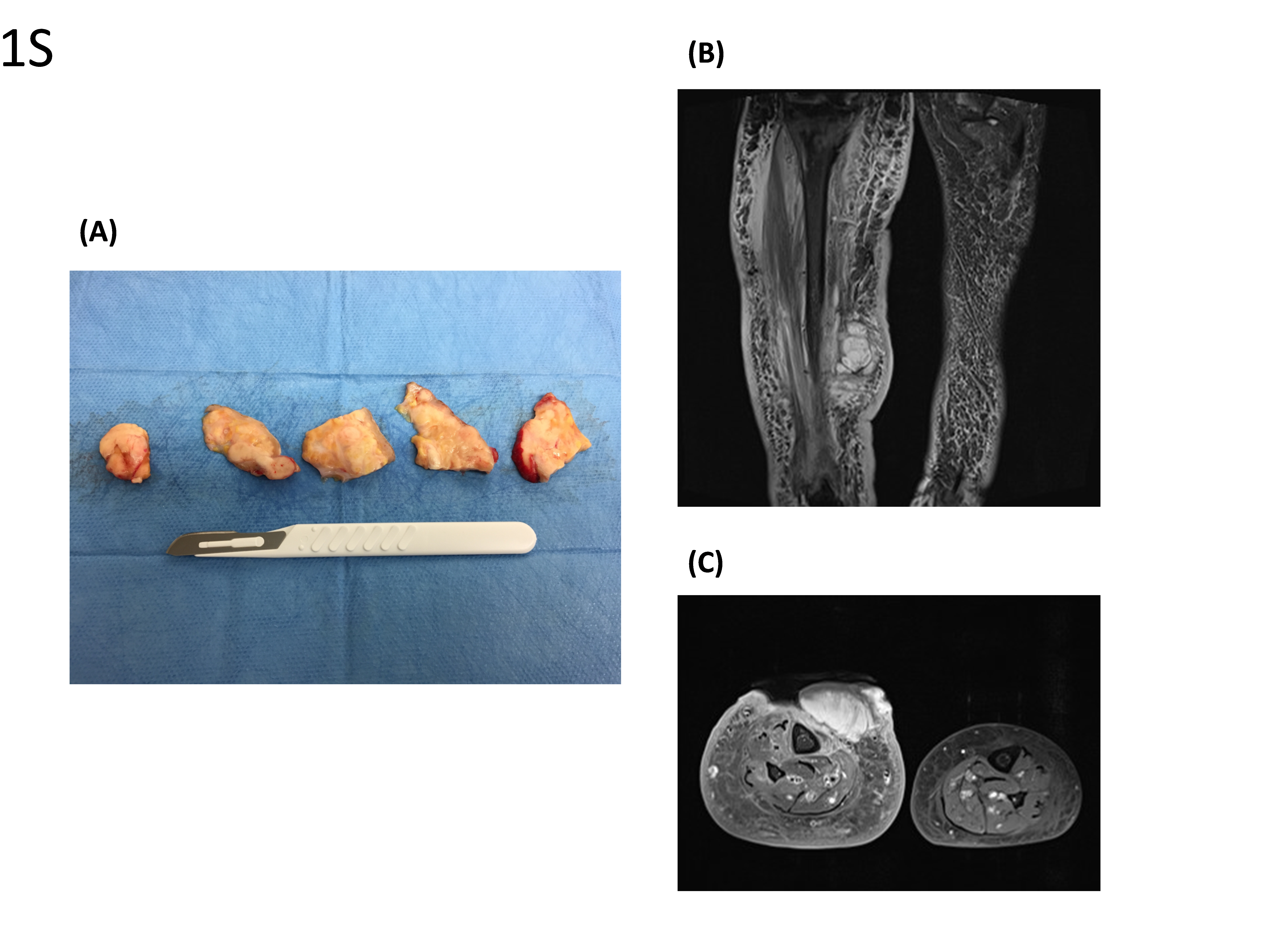
**

**Supplementary Fig. S1** (A) Surgical specimen of UPS patient used for the establishment of S1 primary culture. (B) Coronal post-contrast MRI image showing an expansive formation located in the right thigh presenting an apparent intramuscular infiltration **c)** Axial MRI image of the lesion involving the medial supracondylar of the right thigh.


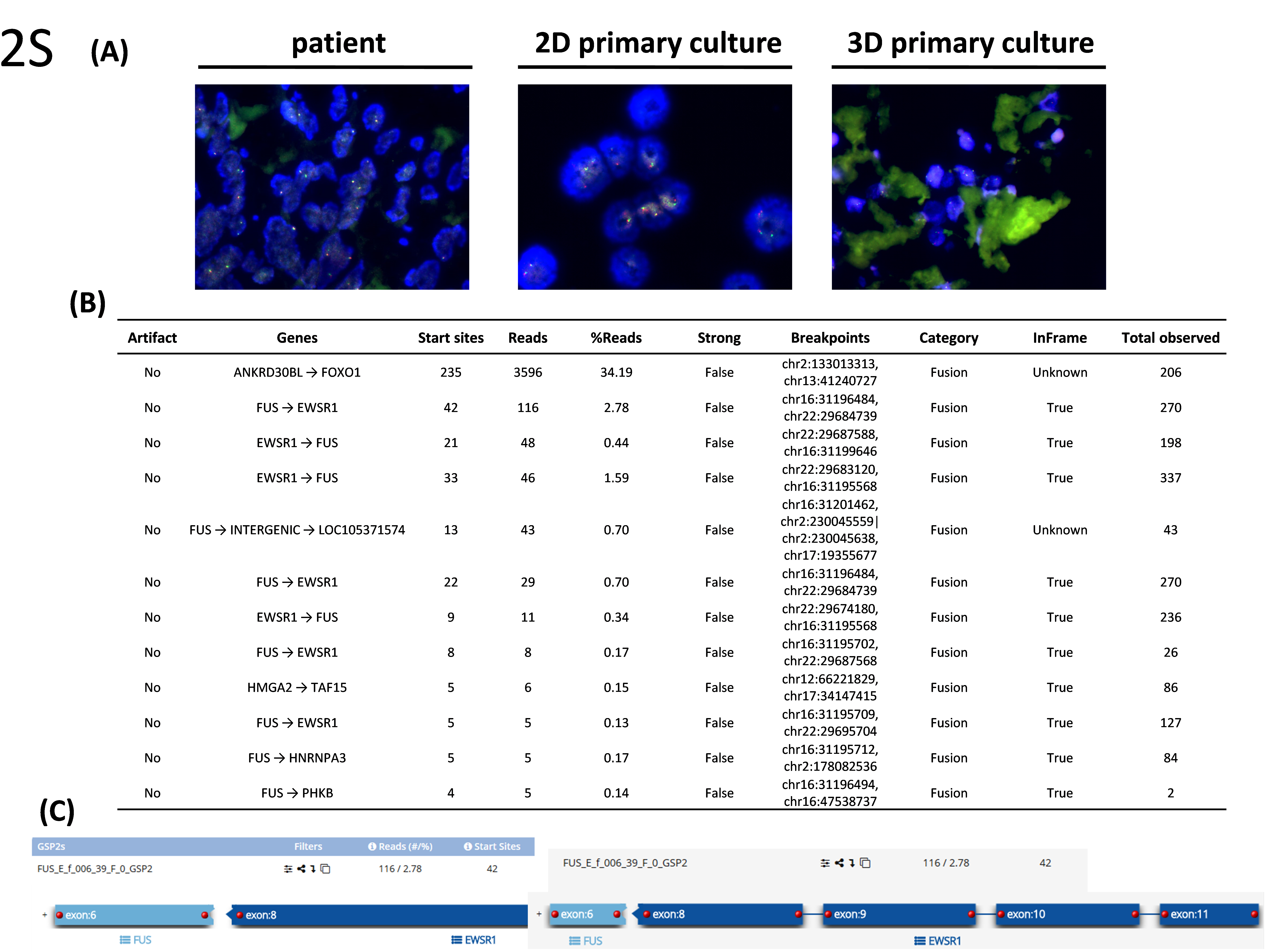


**Supplementary Fig. S2** (A) Representative images of FUS rearrangement analysis of patient specimen, 2D and 3D primary cultures performed by in situ hybridization at 100× magnification [Vysis FUS (16p11) dual color, break apart rearrangement probe kit]. (B) Low confidence fusions detected in S1 patient. No one fusion breakpoint detected passed all the strong evidence filters. c) Representative illustration of FUS-EWSR1 pathognomonic chromosome rearrangements detected in S1 patient.


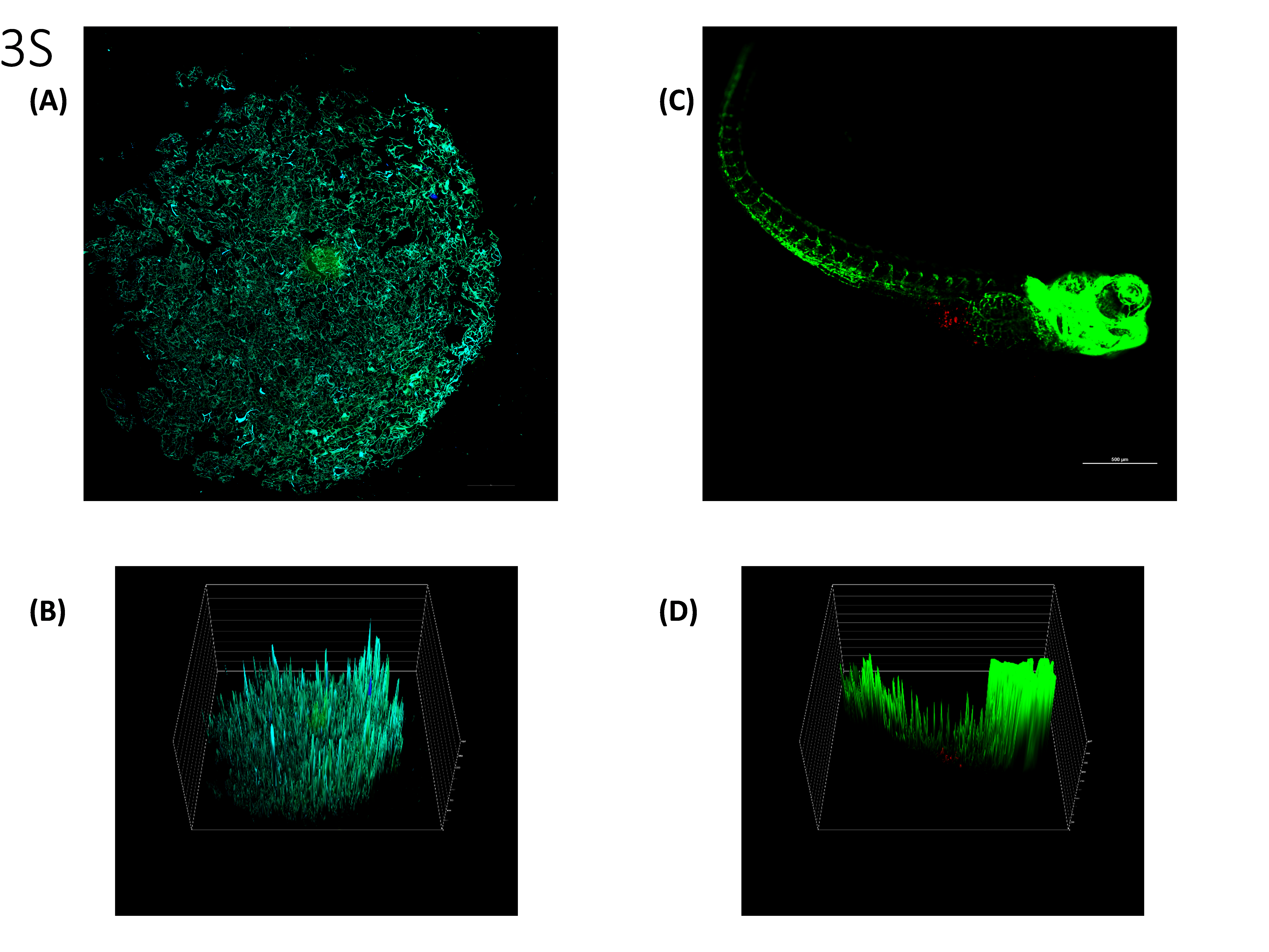


**Supplementary Fig. S3** (A) Representative images of S1 cultured within 3D collagen based scaffold whole section at 24 h and (B) the related intensity surface plot. In green is the autoflorescence of collagen fibers and in blu S1 primary cells stained nuclei. (C) S1 (red spot) xenotranspanted zebrafish embryos at 24h and (D) the related intensity surface plot. the images were acquired with automated inverted Nikon Ti2 confocal microscope.

**
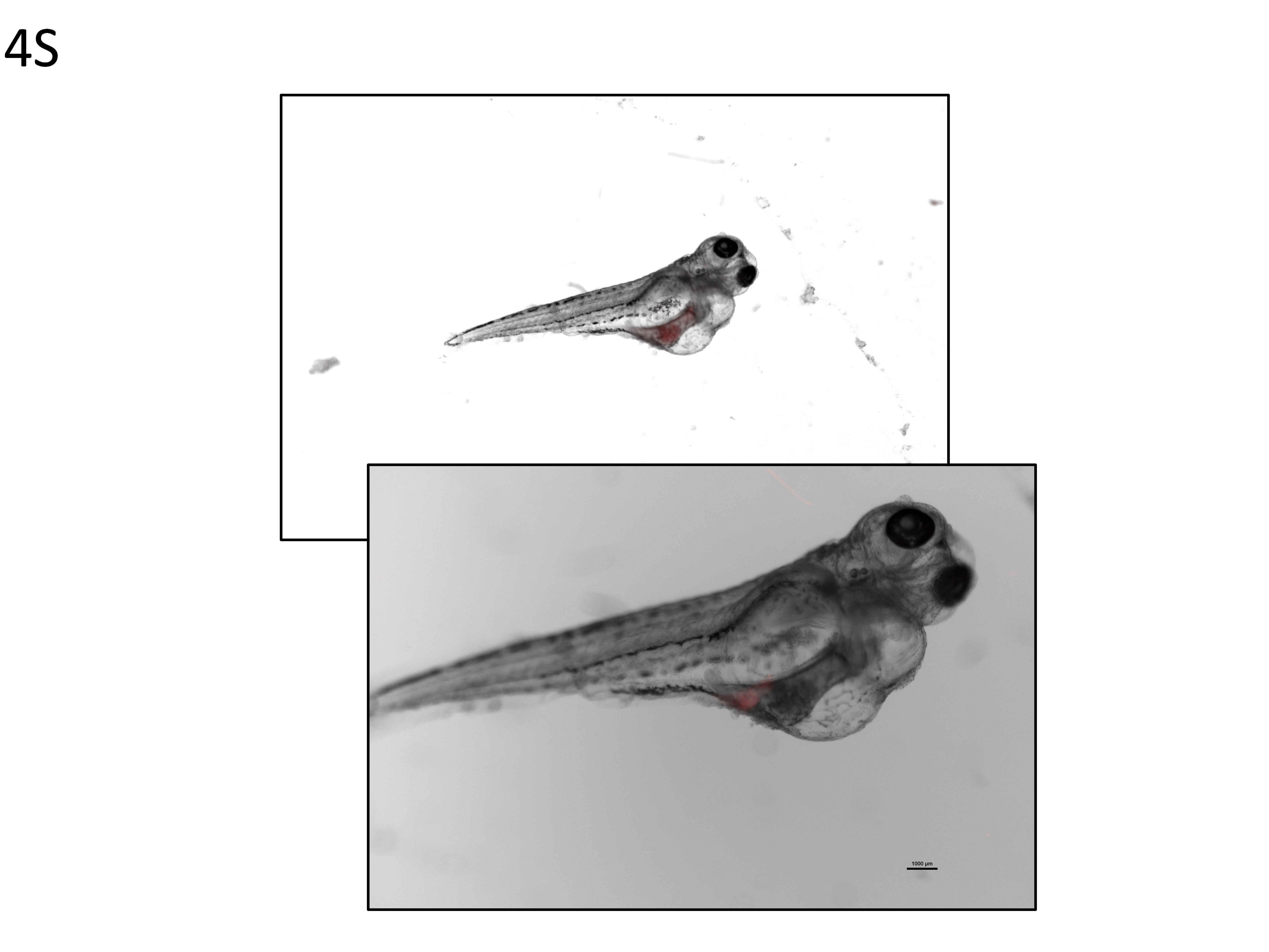
**

**Supplementary Fig. S4** Severe abnormalities including swim bladder deficiency, yolk retention e pericardial edema of zebrafish embryos xenotrasnplanted with S1 primary culture after 72 h exposure with trabectedin, 2x and 4x magnifications.


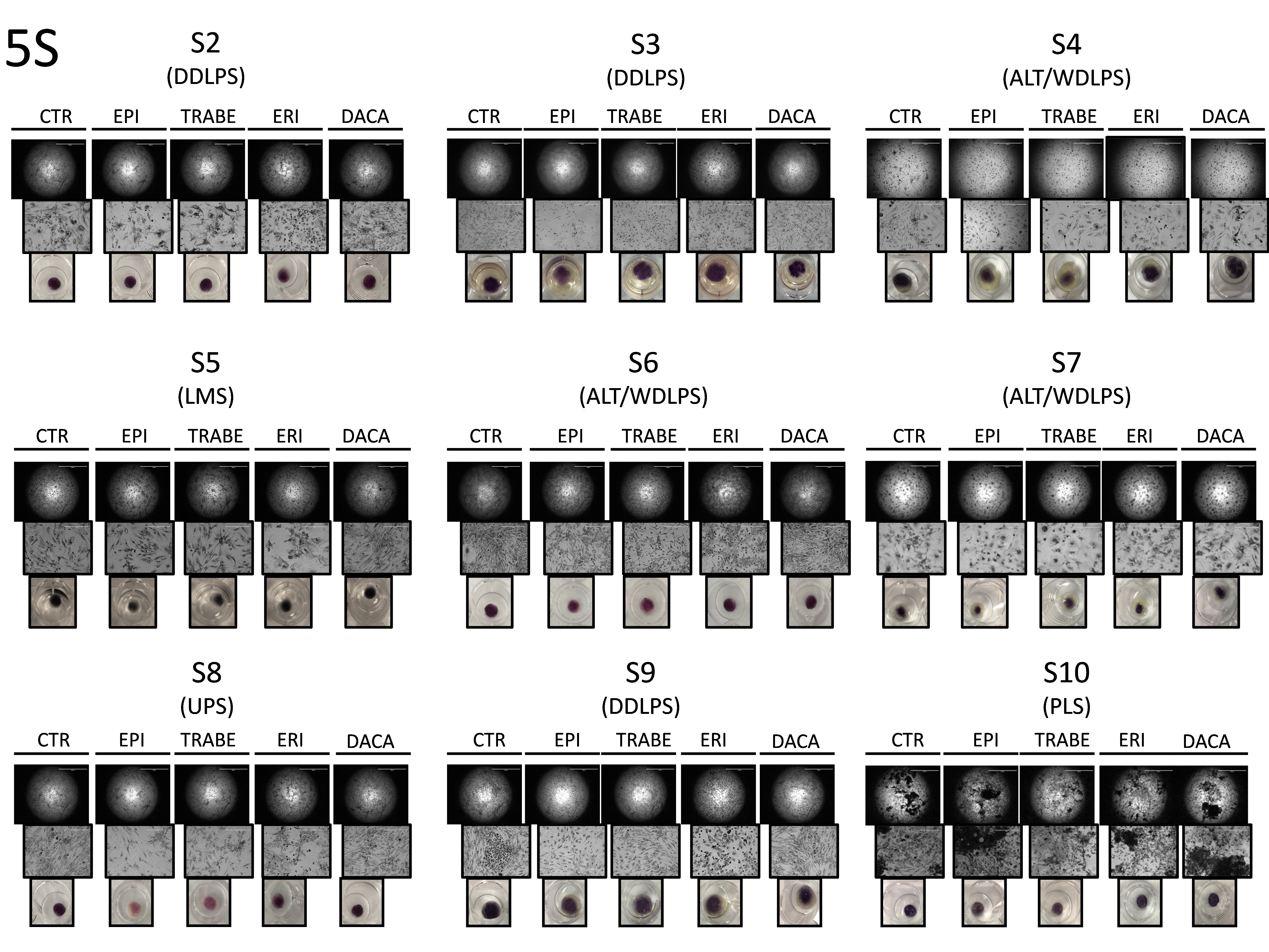


**Supplementary Fig. S5** Representative images of 2D and 3D-collagen based scaffold UPS and L-sarcoma primary culture case series exposed to the tested drugs, 2 x and 10 x magnification.

**
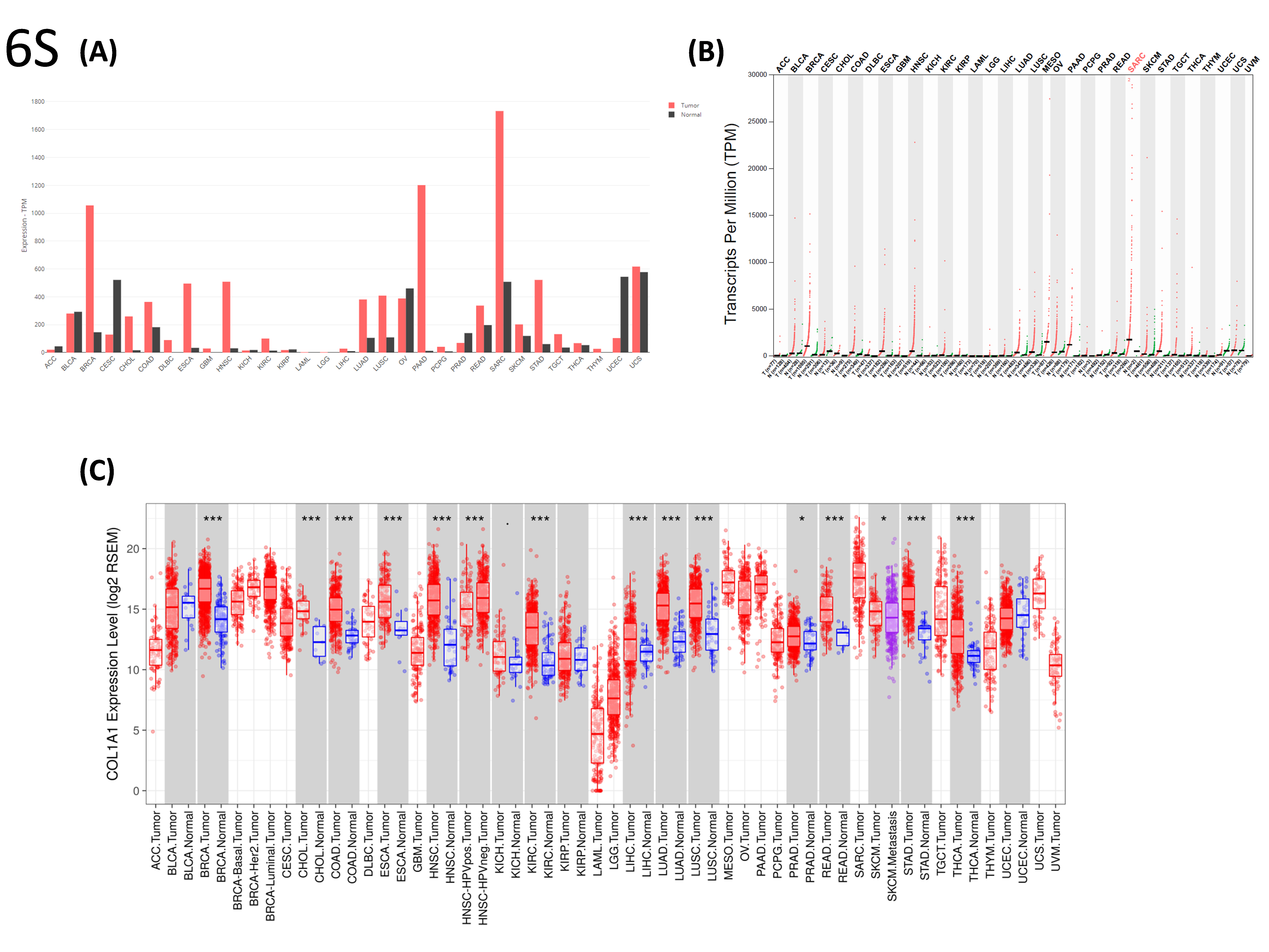
**

**Supplementary Fig. S6** In silico analysis of ECM related gene *col1a1* mRNA expression among tumors and normal tissues. (A) The *col1a1* gene expression profile across all tumor samples and paired normal tissues (bar plot), data expressed as transcripts per million (TPM). (B) The *col1a* expression profile across all tumor samples and paired normal tissues (dot plot), data expressed TPM. (C) The differential expression between tumor and adjacent normal tissues for *col1a1* across all TCGA tumors (box plot). Gepia 2 software was used to obtain a and b panels, TIMER software was used to obtain c panel.

**
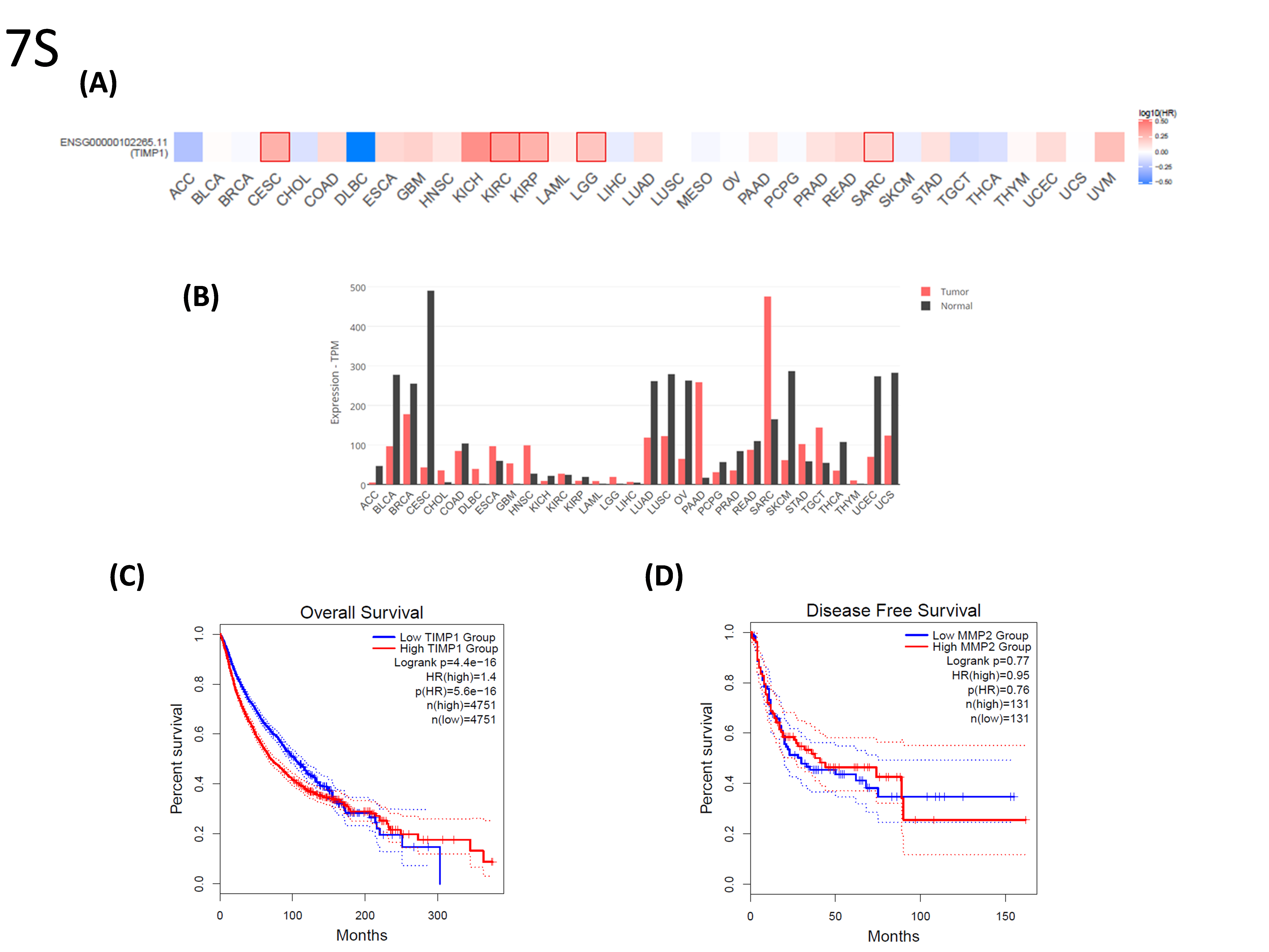
**

**Supplementary Fig. S7** In silico analysis of metallopeptidase inhibitor *timp1* and *mmp2* mRNA expression among tumors and normal tissues. (A) Disease free survival map of *timp1* mRNA expression in multiple cancer types, estimated using Mantel–Cox test. (B) The *mmp2* gene expression profile across all tumor samples and paired normal tissues (bar plot), data expressed as transcripts per million (TPM). (C) Kaplan-Meier curve of overall survival analysis based on the expression status of *timp1* in multiple cancer types. (D) Kaplan-Meier curve of disease free survival analysis based on the expression status of MMP2 in multiple cancer types. Gepia 2 software was used to obtain a-d panels.
