# Supplementary material for: The potential role of the extracellular matrix in the activity of trabectedin in UPS and L-sarcoma: evidences from a patient‐derived primary culture case series in tridimensional and zebrafish models
Source: J Exp Clin Cancer Res. 2021 May 11;40:165. doi: 10.1186/s13046-021-01963-1 (PMC8111914; doi:10.1186/s13046-021-01963-1)
Supplement: Supplementary file 2 — Additional file 2: Supplementary methods. [file 13046_2021_1963_MOESM2_ESM.docx]

**APPENDIX 2 - SUPPLEMENTARY METHODS**

**Supplementary Table S1** Target genes used for RT-PCR analysis

| **Primer** | **Vendor** | **Catalog number** |
| --- | --- | --- |
| *actb* | Thermo Fisher Scientific | Hs_99999903_m1 |
| *hprt* | Thermo Fisher Scientific | Hs_99999909_m1 |
| *mmp2* | Thermo Fisher Scientific | Hs_01548727_m1 |
| *mmp9* | Thermo Fisher Scientific | Hs_00234579_m1 |
| *slug* | Thermo Fisher Scientific | Hs_00161904_m1 |
| *snail* | Thermo Fisher Scientific | Hs_00195591_m1 |
| *tgf-b* | Thermo Fisher Scientific | Hs_00998133_m1 |
| *timp1* | Thermo Fisher Scientific | Hs_99999139_m1 |

**Supplementary Table S2** Chemotherapeutic agents used in pharmacological profiling

| **Drug** | **Vendor** | **Concentration** |
| --- | --- | --- |
| Ifosfamide | Baxter | 100 µM |
| Epirubicin | AccordHealthcare | 2 µg/mL |
| Doxorubicin | Accord Healthcare | 4 µg/mL |
| Trabectedin | PharmaMar | 17 ng/mL |
| Eribulin | Eisai | 371 ng/mL |
| Dacarbazine | Medac Pharma | 8 ug/mL |
| Lenvatinib | Eisai | 0.6 µg/mL |

**Supplementary Table S3** Antibodies used for immunoblotting

| **Antibody** | **Vendor** | **Diluition** |
| --- | --- | --- |
| Casp-3 | Cell Signaling | 1:1000 |
| MDM2 | Cell Signaling | 1:1000 |
| p21 | Cell Signaling | 1:2000 |
| Vinculin | Thermo Scientiﬁc | 1:1000 |

**Histological and immunohistochemical analysis**

Tumor cells morphological features and their distribution was performed through hematoxylin and eosin (H&E) staining. Tissue specimens were washed and paraffin-embedded. Five-µm-thick slices were obtained and stained using standard techniques. MDM2 amplification was assessed by FISH analysis according to manufacturer's instructions (Abbott). FUS chromosomal rearrangement was detected by FISH analysis (Abbott). For monolayer primary cultures, 100.000 cells were cytospinned onto glass slides and downstream analysis were performed following manufacturer’s instructions. For 3D primary cultures, 500.000 cells were cultured for 7 days in collagen-based scaffold and then paraffin-embedded.

**Next-generation sequencing**

NGS analysis were performed as follow: RNA was isolated from FFPE tissue sections using the Qiagen miRNeasy FFPE kit (Qiagen) and purified using the RNeasy MinElute cleanup (Qiagen). Total RNA concentration was measured using the Qubit fluorometer (Thermo Fisher Scientific) and quality checked on the 2100 Bioanalyzer with the RNA 6000 Nano Kit (Agilent Technologies).

Fusion transcript detection was carried out using the commercially available NGS-based fusion panel Archer FusionPlex Sarcoma kit (ArcherDX,) as previously reported.^12^

Libraries preparation was performed according to the manufacturer’s instructions and quality assessed by the 2100 Bioanalyzer (Agilent). Libraries were run on Illumina Miseq (Illumina) platform according to the manufacturer’s instructions. RNA-Sequencing data analysis was processed using the Archer Analysis bioinformatics platform (v 6.2). Fusions that passed all evidence filters were detected.

**Flow cytometry analysis**

A live-dead analysis on 2D or 3D culture was performed after drug exposure. Briefly, primary cells were harvested by trypsinization or by enzymatic digestion in Collagenase type I (Merck Millipore), respectively. To determine cell viability, cells were stained with 50 µM calcein AM and 2 mM ethidium homodimer-1 (Invitrogen). Cell death-related DNA fragmentation was assessed using the In-Situ Cell Death Detection Kit (Roche). The cell suspensions were assayed using BD FACSCanto and FACSDiva software (Becton Dickinson). Samples were run in triplicate and 10,000 events were collected for each replica. Data were the average of three experiments, with errors under 5%.

**Western blot**

Proteins were isolated from 3D S1 using a MPER Buffer supplemented with a 0.5% protease inhibitor cocktail. BCA protein assay kit (Thermo Scientiﬁc) was used to determine protein content. An equal amount of protein from each sample was separated on Criterion™ Precast Gel Tris-HCl (Biorad) and transferred to polyvinylidene ﬂuoride membranes (Millipore Corporation). After blocking the membranes were incubated with primary antibody and with horseradish peroxidase-conjugated secondary antibody (see complete list of antibody in Supplementary Table S3). Densitometric analysis of proteins bands was performed with Quantity One software version 4.6.9 (BioRad).

**Zebrafish husbandry**

Zebrafish husbandry protocols were performed in conformity with the Directive 2010/63/EU and in compliance with local animal welfare regulations (authorization n. prot. 18311/2016; released by the “Comune di Meldola”, 09/11/2016). AB wild type strain fertilized eggs were obtained and stored according to Kimmel et al.^21^ Before any manipulation, the embryos were anesthetized in 0,02 % tricaine solution.

***In silico* analysis**

Gene expression profiling interactive analysis 2 (GEPIA 2, http://gepia2.cancer-pku.cn/#general) and Tumor IMmune Estimation Resource (TIMER, https://cistrome.shinyapps.io/timer/) were used for differential expression analysis of COL1A1 in tumor and normal tissue from various cancers.
