# Supplementary material for: The potential role of the extracellular matrix in the activity of trabectedin in UPS and L-sarcoma: evidences from a patient‐derived primary culture case series in tridimensional and zebrafish models
Source: J Exp Clin Cancer Res. 2021 May 11;40:165. doi: 10.1186/s13046-021-01963-1 (PMC8111914; doi:10.1186/s13046-021-01963-1)
Supplement: Supplementary file 3 — Additional file 3: Supporting information. [file 13046_2021_1963_MOESM3_ESM.docx]

**APPENDIX 3 - SUPPORTING INFORMATION**

**Patient clinical history**

S1 was a 81 year-old female patient with a diagnosis of right leg UPS. The radiological imaging, including a MRI showed the presence of an expansive oval neoformation measuring 10 x 7 x 5.5 cm with multiple satellite nodules without evidence of metastatic disease (Supplementary Fig. S1). The biopsy was performed at another Center with the histological diagnosis of pleomorphic liposarcoma. The Multidisciplinary Board indicated the surgical removal of the lesion in the right leg. On March 2018 the patient underwent a surgery of the lesion with the amputation of the leg; the pathology revealed an ulcerated and necrotic neoformation measured 18 x 8 cm and consisted of cells from high-grade polymorphic UPS with positive surgical margins. The radiological evaluation after the surgery didn’t showed macroscopical metastatic disease. The multidisciplinary evaluation suggested adjuvant radiotherapy treatment.

S2 was a 83-year-old female patient with a diagnosis of DDLPS of the right limb treated with surgery in 2011; the pathologic report revealed high grade DDLPS, with infiltrated surgical margins. A re-excision of the lesion was performed with no evidence of residual disease. Subsequently she completed 50 Gy of local radiotherapy. Three years later, during the follow up, there was the evidence of local recurrence. For this reason the patient underwent a radical surgery in another Center and at that time information is lacking about the surgery and the pathology. In March 2016, an integrated [(18)F]fluorodeoxy glucose positron emission tomography-computed tomography (PET CT) scan revealed recurrence of disease in the muscle of the right gluteus close to the primary tumor with the standard uptake value (SUV) max of 3.4. Moreover there was one nodule in the lung without pathological accumulation. After multidisciplinary evaluation, the patient underwent to surgical resection of the disease in the gluteus; the pathological report showed the presence of high grade DDLPS with MDM2 gene amplification. Surgical margins were positive. The patient performed local radiotherapy that was terminated in October 2016 (50 Gy). In June 2017 an additional PET CT scan revealed the presence of new local recurrence of about 20x15 mm and the appearance of three pulmonary metastasis. The Multidisciplinary Board suggested a biopsy in the lung that was not diagnostic; the patient refused another biopsy. The pulmonary surgical was not indicated. The patient started a first line of chemotherapy with trabectedin according to the Italian Sarcoma Group (ISG) clinical study Efficacy Study on Trabectedin in Retroperitoneal Leiomyosarcoma and Well Differentiated/Dedifferentiated Liposarcoma (TRAVELL). The patient had a local disease progression as well as in a single nodule in the lung. In June 2018 performed radiotherapy in the lung metastases. The patient died in January 2019.

S3 was a 72-year-old female patient with recurrences from liposarcoma of the limb. She had the first diagnosis of well differentiated liposarcoma of the right limb in 1986 in Romania. Subsequently the patient had several local recurrences surgically removed in the same Center. The most recent recurrence occurred in 2012 and was operated at Rizzoli Institute in Bologna.

In February 2019 the patient underwent to surgical resection of the another recurrence of liposarcoma that was described in the histology report as liposarcoma dedifferentiated with prevalent low grade dedifferentiated areas and negative surgical margins. The MDM2 gene was amplified.

The CT scan after surgery was negative and the Muldisciplinary Board indicated to continue the follow up that is still negative per recurrence of distant and local disease.

S4 was a 61-year-old female patient referred to our center for limb liposarcoma. In May 2019 the patient underwent to surgery of the lesion in the left thigh. The histology showed the presence of well differentiated liposarcoma. Evaluation of the MDM2 gene was amplified with FISH method.

The post-surgery imaging revealed no evidence of the tumor. The Multidisciplinary Board suggested a follow up after surgery. The patient is still in follow up evaluation.

S5 was a 77-year-old female patient referred to our center for retroperitoneal leiomiosarcoma. The surgical resection of the tumor was performed in September 2019 and the histology revealed a grade 2 leiomiosarcoma of the retroperitoneum. The imaging post-surgery exhibited no evidence of oncological disease. The Multidisciplinary Board suggested a follow up after surgery. The patient is going well in follow up evaluation.

S6 was a 71-year-old female patient with a diagnosis of well differentiated liposarcoma of the retroperitoneum. The surgery was performed in April 2019 and consisted of en bloc resection of the lesion and the right colon, the anterior portion of VI hepatic segment and the right kidney. The histology showed low grade well differentiated liposarcoma of the retroperitoneum. The radiological evaluation post-surgery exhibited no evidence of oncological disease. The multidisciplinary board suggested a follow up after surgery that is still ongoing.

S7 was a 56-year-old male patient with a diagnosis of well differentiated liposarcoma of the extremity. In January 2020 patient was referred at our Institute after a biopsy of her right axillary mass revealed cells from atypical lipomatous tumor/well differentiated liposarcoma. Subsequently imaging exams were performed including MRI of the axilla and CT angiogram for local vascular assessment. Total body CT scan showed no evidence of metastasis. The surgical asportation of the lesion underwent in February 2020 and the pathology report confirmed atypical lipomatous tumor/well differentiated liposarcoma involving the right proximal arm and omolateral axilla with negative margins. The post-surgical radiological evaluation is ongoing.

S8 was a 78-year-old female patient with a diagnosis of undifferentiated pleomorphic sarcoma of the left thigh. In July 2019 the patient underwent the surgical removal of the 6 cm neoformation in the left thigh; the pathology report revealed the presence of cells from undifferentiated pleomorphic sarcoma. The surgical margins were negative. The CT scan and MRI after the surgery were negative. The multidisciplinary board suggested adjuvant radiotherapy that the patient completed in January 2020. The subsequent radiological evaluation didn’t show recurrence of disease. The follow up of the patient is still ongoing without evidence of recurrence.

S9 was a 57 year-old female patient with a diagnosis of dedifferentiated liposarcoma of the abdomen. The patient was referred to our center after the surgical removal of the abdominal formation in the left iliac region. The histology of surgical specimen revealed grade 3 dedifferentiated liposarcoma of 9 cm with the amplification of MDM2; the muscular margins were positive. The CT scan evaluation after surgery was negative for local and distant recurrences. The multidisciplinary board suggested adjuvant treatments with chemotherapy and radiotherapy that the patient refused. After three months the radiological evaluation showed the presence of two metastases in the lung.

S10 was a 64 year-old female patient with a diagnosis of extremity pleomorphic liposarcoma. In anamnesis the patient underwent to nephrectomy in 1995 for clear cell renal carcinoma.

The patient was referred to our center with the appearance of formation in the right thigh of about 6.5 cm. The biopsy revealed cells from high grade pleomorphic liposarcoma. The multidisciplinary board indicated the surgical removal of the formation that was performed in December 2019. The histology report confirmed the presence of high grade pleomorphic liposarcoma of 7 cm; the surgical margins were positive. The postoperative CT scan showed the presence of two nodules in the pancreatic head suggestive for primary lesions. The eco-endoscopy with biopsy showed the presence of cells from clear cell renal carcinoma in the pancreatic nodules. The patient started first line treatment for the renal carcinoma that is ongoing.

**Establishment of UPS and L-sarcoma primary cultures**

S2

Macroscopic evaluation of surgically-resected tumor tissue revealed a compact fragment of well delimited fibroadipose and skeletal muscle tissue weighting 143 g and measuring 10 x 8 x 4 cm. The specimen included two roundish white-yellowish and gray-white nodules of 2.5 cm and 1 cm of major axis. Hematoxylin and eosin-stained tumor tissue was reviewed by an experienced sarcoma pathologist who reported the of a DDLPS with positive surgical margins. MDM2 gene amplification analysis carried out by FISH assay was positive, supporting the diagnosis of high grade DDLPS. Cytomorphologic features analysis of patient-derived S2 cells reviewed by an experienced sarcoma pathologist confirmed the establishment of DDLPS primary culture with a proportion of DDLPS cells of 20%.

S3

Macroscopic evaluation of surgically-resected tumor tissue revealed a fragment consisting of skin and hypodermic dermal tissue weighting 393 g and measuring 15 x 11 x 8 cm. The skin showed cicatricial area of 3 x 2.5 cm and when cut a yellowish polylobate newborn formation of 13 cm of major axis was present. Hematoxylin and eosin-stained tumor tissue confirmed a diagnosis of DDLPS with prevalent low grade dedifferentiation. Surgical margins were negative. Cytomorphologic features analysis of patient-derived S3 cells reviewed by an experienced sarcoma pathologist confirmed the establishment of DDLPS primary culture with a proportion of DDLPS cells of 10%.

S4

Macroscopic evaluation of surgically-resected tumor tissue revealed an adipose nodule weighting 595 g and measuring of 20 x 15 x 3.5 cm. Hematoxylin and eosin-stained tumor tissue showed an intramuscular adipocyte proliferation with lipoblasts, focal lymphocyte infiltrate and rare myxoid areas. The diagnosis was of ALT/WDLPS. MDM2 gene amplification analysis carried out by FISH assay was positive, supporting the diagnosis of high grade ALT/WDLPS. Cytomorphologic features analysis of patient-derived S4 cells reviewed by an experienced sarcoma pathologist confirmed the establishment of ALT/WDLPS primary culture with a proportion of ALT/WDLPS cells of 15%.

S5

Macroscopic evaluation of surgically-resected tumor tissue revealed: distal pancreas 6 cm of length, spleen 10 cm, left kidney 12 cm with adrenal gland. In fatty tissue located between the kidney and pancreas was a grayish nodular neoformation with a stretch-elastic consistency of 10 cm of major axis. Hematoxylin and eosin-stained tumor tissue showed a grade 2 LMS according to the *Fédération Nationale des Centres de Lutte Contre le Cancer* (FNCLCC) with rare outbreaks of ischemic necrosis. The neoplasm reaches the capsule, without however going beyond it (marginal resection). Tumor cells were positive for SMA and desmin. Pancreas, spleen, left kidney and left adrenal gland free of neoplastic infiltration. MDM2 amplification was investigated using Vysis MDM2 / CEP12 Dual Color FISH Probe Kit probe. 100 nuclei in interphase were examined and MDM2 gene amplification was absent (MDM2/CEP 12 = 1.2). Surgical margins were negative. Cytomorphologic features analysis of patient-derived S5 cells reviewed by an experienced sarcoma pathologist confirmed the establishment of LMS primary culture with a proportion of LMS cells of 50%.

S6

Macroscopic evaluation of surgically-resected tumor tissue revealed: a terminal ileal loop for a total of 4 cm of length, ileocecal valve, blind and ascending colon for a total of 24 cm of length, cecal appendix for a total of 7 cm of length, liver segment 3.5 x 1 x 6 cm, kidney 11 cm major axis with ureter of 6 cm. Lipomatous neoformation of 10 cm major axis firmly attached to the liver, colon and kidney. Hematoxylin and eosin-stained tumor tissue showed a ALT/WDLPS lesion with a low-grade focal area of ​​dedifferentiation, extended to the tissue mesenteric up to near the colon muscle wall, to the peri-renal adipose tissue up to renal capsule and tenaciously adhered to the Glissonian liver margin and a fragment of muscle tissue striatum. Surgical margins were negative. Cytomorphologic features analysis of patient-derived S6 cells reviewed by an experienced sarcoma pathologist confirmed the establishment of ALT/WDLPS primary culture of ALT/WDLPS cells of 10%.

S7

Macroscopic evaluation of surgically-resected tumor tissue revealed a lipomatous mass weighting 650 g and measuring 15 x 14 x 4 cm with flap of skin and subcutaneous tissue of 13 cm sutured to the mass. To the cut the lesion showed areas of soft texture. Hematoxylin and eosin-stained tumor tissue showed a ALT/WDLPS. The skin and subcutaneous tissues were free from neoplastic infiltration as the two lymph nodes that have been founded. Surgical margins were negative. Cytomorphologic features analysis of patient-derived S7 cells reviewed by an experienced sarcoma pathologist confirmed the establishment of ALT/WDLPS primary culture with a proportion of ALT/WDLPS cells of 25%.

S8

Macroscopic evaluation of surgically-resected tumor tissue revealed cutaneous lozenge of 13 x 7 cm with cystic area with a blood content of 6 cm of major axis. The edges of the cyst were made of brownish tissue. Hematoxylin and eosin-stained tumor tissue showed a malignant neoplasm from spindle cells and large pleomorphic cells and deposits of hemosiderin. The neoplasm was located subcutaneously and was totally contained within the deep margin and within the lateral margins of surgical resection. A bland and focal positivity was established for SMA, while CD31, CD34, S100, cytokeratins, desmin, CD117 were negative. The diagnosis was of undifferentiated pleomorphic sarcoma. Surgical margins were negative. Cytomorphologic features analysis of patient-derived S8 cells reviewed by an experienced sarcoma pathologist confirmed the establishment of UPS primary culture with a proportion of UPS cells of 40%.

S9

Macroscopic evaluation of surgically-resected tumor tissue revealed a localized neoformation in left iliac fossa. The mass weighting 375 g and measuring 13 x 11 x 8 cm partly was covered by peritoneum hyperemic and with attached muscle segment. The mass was of hard consistency, whitish color and collated appearance measured 9 x 8 cm. Hematoxylin and eosin-stained tumor tissue showed large areas of necrosis and morphological aspects rather various with spindle cell areas and epithelioid areas. MDM2 amplification was investigated using Vysis MDM2 / CEP12 Dual Color FISH Probe Kit probe. 100 nuclei in interphase were examined and MDM2 gene amplification was present (MDM2/CEP 12 = 2). The diagnosis was of dedifferentiated liposarcoma. The excision appears complete. On the deep muscular plane, the lesion was less than 1 mm from the margin. Cytomorphologic features analysis of patient-derived S9 cells reviewed by an experienced sarcoma pathologist confirmed the establishment of DDLPS primary culture with a proportion of DDLPS cells of 35%.

S10

Macroscopic evaluation of surgically-resected tumor tissue revealed a skeletal muscle segment 9 cm long with a lipomatous neoplasm of 7 cm in major axis yellowish with whitish areas. Hematoxylin and eosin-stained tumor tissue showed a sarcomatous proliferation consisting of markedly atypical elements with numerous mitoses and areas rich in lipoblasts. MDM2 amplification was investigated using Vysis MDM2 / CEP12 Dual Color FISH Probe Kit probe. 100 nuclei in interphase were examined and MDM2 gene amplification was absent (MDM2/CEP 12 = 0.85). Based on morphological and molecular analysis the diagnosis was of pleomorphic liposarcoma. Surgical margins were negative. Cytomorphologic features analysis of patient-derived S10 cells reviewed by an experienced sarcoma pathologist confirmed the establishment of PLS primary culture with a proportion of PLS cells of 25%.

***col1a1* gene is upregulated in soft tissue sarcoma compared to other tumors**

In order to further investigate if there is a correlation between ECM related components and trabectedin mechanism of action an *in silico* analysis of *col1a1* mRNA expression among tumors and normal tissues was performed. The results clearly showed an upregulation of *col1a1* gene in various tumor tissue (Supplementary Fig. S6). Among all, STS, breast invasive carcinoma and pancreatic adenocarcinoma showed the higher upregulation of *col1a1* gene. Moreover, in order to support the previous data an *in silico* analysis of *timp1* and *mmp2* was performed (Supplementary Fig. S7). The results showed a significant positive role of *timp1* in STS disease free survival and in solid tumors overall survival. Moreover *mmp2* resulted strongly upregulated in STS compared various tumors and exhibited a significant negative role in overall survival in solid tumors. The results were confirmed using two different software (GEPIA 2 and TIMER).
